# Beyond the dichotomy between controlled and naturalistic paradigms: modeling psychological constructs in cognitive neuroscience

**DOI:** 10.3389/fpsyg.2026.1881045

**Published:** 2026-07-23

**Authors:** Tomoya Nakai

**Affiliations:** Graduate School of Information Science and Technology, The University of Tokyo, Tokyo, Japan

**Keywords:** controlled paradigm, encoding model, naturalistic paradigm, NeuroAI, psychological construct

## Abstract

Cognitive neuroscience aims to link psychological constructs to brain activity, but this goal depends critically on how such constructs are operationalized. Conventional controlled paradigms define constructs through task contrasts or parametric manipulations, providing interpretability by restricting the range of stimuli, behavioral responses, and task demands. Naturalistic paradigms instead use complex, temporally extended stimuli such as movies, narratives, and music, enabling brain activity to be modeled in relation to continuous, multidimensional features of real-world inputs. With the rise of intersubject correlation analyses, encoding models, and NeuroAI approaches, naturalistic neuroimaging has expanded from the characterization of shared brain activity across participants to high-dimensional predictive modeling using features derived from stimuli, behavior, and artificial neural networks. However, this shift does not eliminate theoretical assumptions; it relocates them from task design to feature-space selection, model architecture, and model evaluation. This review argues that controlled and naturalistic paradigms should be treated as complementary strategies rather than competing alternatives. Integrating hypothesis-guided task design with computational modeling may extend neuroimaging beyond passive perception toward active cognition, including reasoning, decision making, and mathematical problem solving. Such an integrative framework can provide a broader basis for testing, refining, and potentially redefining psychological constructs through their relationships to neural and computational representations.

## Introduction

Cognitive neuroscientists have long sought to understand how various psychological constructs are represented in the brain. Constructs such as intelligence, working memory, motivation, and attention were originally introduced in the field of psychology and cognitive science to explain human behavior and cognition (Cronbach and Meehl, 1955; Danziger, 1997). The development of noninvasive functional neuroimaging techniques, particularly functional magnetic resonance imaging (fMRI), has encouraged the view that these constructs can be grounded in measurable patterns of brain activity.

At the same time, substantial criticisms have been raised against the *reification* of psychological constructs through neuroimaging. One central concern is that individual brain regions are often involved in multiple cognitive processes, making it difficult to infer mental functions directly from neuroimaging data and challenging simplistic one-to-one mappings between brain regions and psychological constructs (Poldrack, 2006, 2010a). This concern has motivated efforts to formalize the relationships among psychological constructs, experimental tasks, and neural representations through cognitive ontologies (Poldrack and Yarkoni, 2016).

Related philosophical work has further emphasized that successful integration between psychology and neuroscience requires stable constructs and explicit accounts of how cognitive capacities, tasks, and neural mechanisms are related (Sullivan, 2016; Francken et al., 2022). This issue is also highlighted by approaches that question whether intervening psychological constructs are always necessary for linking behavior and brain activity (Shulman and Rothman, 2019), or whether researcher-defined psychological domains align with the data-driven structure of large-scale neuroimaging results (Beam et al., 2021).

This issue is further complicated by the status of psychological constructs as explanatory variables. Such constructs differ from physically measurable stimulus properties, such as sound pressure, luminance, or the orientation of a line segment, which can be defined in terms of physical or geometric properties of the stimulus. Constructs such as intelligence, working memory, motivation, or attention, by contrast, are theoretical entities whose measurement depends on operational definitions and construct validation (Cronbach and Meehl, 1955; Strauss and Smith, 2009; Clark and Watson, 2019). In neuroimaging analyses, however, these quantities are often treated in formally similar ways: once operationalized, they can all be entered into a statistical or computational model as variables to explain variation in brain activity.

**Figure 1:**
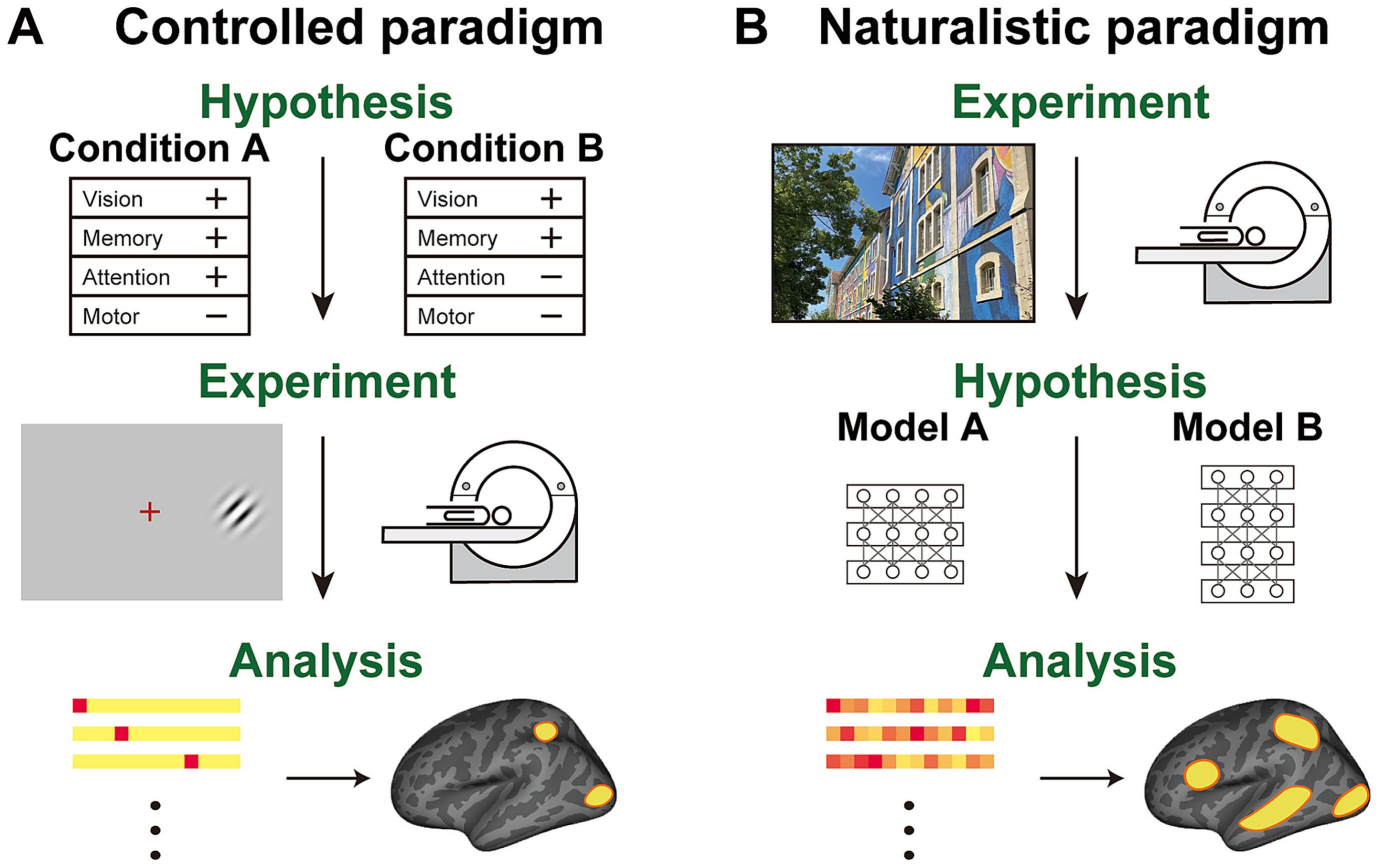
Hypothesis placement in controlled and naturalistic paradigms. **(A)** In controlled paradigms, hypotheses are embedded before data collection through the design of task conditions, and brain activity is analyzed by comparing predefined conditions. **(B)** In naturalistic paradigms, rich stimuli are presented first, and hypotheses are introduced during analysis through the choice of computational models or feature spaces.

This formal equivalence may obscure an important assumption. An experimentally defined variable can be interpreted as an index of a psychological construct only when the task or stimulus successfully elicits, manipulates, or measures that construct (Poldrack et al., 2011; Turner and Laird, 2012; Poldrack and Yarkoni, 2016; Francken et al., 2022). For cognitive neuroscientists, a major methodological challenge has therefore been to design such tasks and stimuli so that the effect of the target construct can be isolated from those of other, non-target processes. This is the basic logic of experimental control in (task-based) *controlled paradigms* (Figure 1A): by carefully matching conditions in all aspects except for the construct of interest, researchers aim to attribute brain activity to that construct.

This logic, however, has been increasingly challenged by the rise of computational approaches and the growing use of *naturalistic paradigms* (Sonkusare et al., 2019; Nastase et al., 2020) (Figure 1B). Rather than isolating a single psychological construct through controlled contrasts, naturalistic paradigms use richer and more ecologically valid stimuli, such as narratives, movies, or social interactions (Hasson et al., 2004; Wilson et al., 2008; Stephens et al., 2010; Lerner et al., 2011; Nishimoto et al., 2011; Dikker et al., 2014; Misaki et al., 2021). These paradigms shift the focus from comparing discrete experimental conditions to modeling how brain activity varies with continuously changing features of stimuli, behavior, and internal states, often using encoding models or related computational approaches (Naselaris et al., 2011; Kriegeskorte and Douglas, 2019). In this sense, naturalistic paradigms should not be viewed merely as ecologically valid experiments; rather, they change the way psychological constructs are introduced into neuroimaging studies.

In this article, we first review the basic logic underlying conventional controlled paradigms, with particular attention to how psychological constructs are operationalized through experimental contrasts. We then clarify how naturalistic paradigms depart from this logic, especially in their treatment of stimuli, tasks, and explanatory variables. Next, we discuss how computational approaches, including encoding models and artificial neural network (ANN)-based models, have expanded the scope of naturalistic neuroimaging. Finally, we consider the limitations of naturalistic paradigms and argue for a broader framework that integrates controlled and naturalistic approaches.

### Controlled paradigm

Conventional functional neuroimaging studies have widely relied on the controlled paradigm, often based on the logic of *cognitive subtraction* originally developed in mental chronometry (Donders, 1868/1969; Sternberg, 1969; Poldrack, 2010b) and later adopted in early positron emission tomography studies to map cognitive functions onto brain regions (Petersen et al., 1988; Posner et al., 1988). In this approach, a target task is designed to strongly engage a particular psychological construct, whereas a control task is designed to engage that construct to a lesser degree while otherwise matching the target task in terms of non-target processes. The resulting activity difference is interpreted as reflecting the neural correlates of the target construct. Such conditions are typically modeled as discrete categories, often using separate indicator variables for each task or stimulus condition, so that brain activity is modeled as a function of qualitatively defined task states rather than continuous psychological or stimulus dimensions.

Alternatively, psychological constructs can be represented through continuous variables. Parametric designs model brain activity as functions of experimental parameters rather than as simple condition differences (Cohen, 1997; Büchel et al., 1998). For example, cognitive load, reaction time, and subjective ratings can be used as quantitative indices of task demands, processing time or effort, and emotional states, respectively (Braver et al., 1997; Phan et al., 2004; Taylor et al., 2014). More generally, model-based fMRI extends this approach by using computationally derived latent variables, such as value or prediction error, as quantitative regressors of brain activity (O’Doherty et al., 2007).

It is important to note that the distinction between controlled and naturalistic paradigms is orthogonal to the distinction between univariate and multivariate analyses. Although multivariate analyses examine distributed activity patterns rather than mean activation within a region (Haxby et al., 2001; Haynes and Rees, 2006), they remain part of a controlled paradigm when they compare experimentally defined task conditions. The same logic applies to other brain activity measurements, including functional connectivity and gray-matter volume (Mechelli et al., 2005; Friston, 2011). What differs across these approaches is not how the psychological construct is operationalized, but the brain measure to which the construct is related.

A crucial feature of controlled paradigms is that the relevant explanatory variables are specified in advance by the researcher. Psychological constructs are operationalized through tasks or stimuli designed to elicit them, and brain activity is tested against these predefined variables. The interpretation of the resulting brain–construct relationship depends on whether the task successfully elicits the intended construct and whether participants perform it in the manner assumed by the experimenter. Controlled paradigms address this challenge by reducing the degrees of freedom in the experiment: tasks and stimuli are simplified, nuisance factors are matched or eliminated, and participants are constrained to perform a limited set of operations. This reduction of variability allows researchers to test specific hypotheses under conditions in which alternative explanations are minimized. In this sense, controlled paradigms obtain interpretability by restricting the space of possible stimuli, behaviors, and strategies.

Thus, controlled paradigms are well suited for testing predefined hypotheses about specific constructs, but they are less suited for discovering how multiple constructs jointly arise, interact, or vary in more complex situations. This limitation becomes particularly important when the goal is not merely to localize a predefined construct, but to understand how cognitive dimensions are organized in rich, multidimensional environments.

### Naturalistic paradigm

Naturalistic paradigms have gained increasing attention for measuring brain activity under conditions closer to everyday cognition (Sonkusare et al., 2019; Nastase et al., 2020; Saarimäki, 2021; Zhang et al., 2021), while naturalistic stimuli had already been discussed in neurophysiology as a complement or alternative to well-controlled artificial stimuli (Creutzfeldt and Nothdurft, 1978; Felsen and Dan, 2005; Rust and Movshon, 2005). In these paradigms, participants typically watch movies, listen to spoken narratives, or experience other temporally extended stimuli while their brain activity is recorded, often with minimal explicit task demands (Hasson et al., 2004, 2010). Because participants are usually asked simply to attend to the stimulus, the need for extensive task training or complex behavioral instructions is reduced. At the same time, the stimuli used in naturalistic paradigms are generally richer than those used in controlled paradigms, and their features are often left intact rather than being experimentally manipulated in advance (Sonkusare et al., 2019; Nastase et al., 2020).

Early applications of naturalistic paradigms often relied on intersubject correlation (ISC) analysis to characterize shared brain activity during passive viewing or listening. In ISC, brain activity time series elicited by the same stimulus are compared across participants, and brain activity that are reliably correlated across individuals are interpreted as stimulus-driven patterns shared by the group. This approach was first demonstrated in movie-viewing fMRI, where natural visual scenes evoked synchronized cortical activity across viewers (Hasson et al., 2004), and was later extended to spoken narratives, verbal communication, and music listening (Stephens et al., 2010; Dikker et al., 2014; Trost et al., 2015). ISC has since been formalized as a general framework for measuring shared stimulus-driven responses during naturalistic stimulation (Hasson et al., 2010; Nastase et al., 2019).

Encoding models provide a distinct framework from ISC for relating brain activity to the feature spaces underlying complex stimuli in naturalistic paradigms (Naselaris et al., 2011). In this approach, different types of features can be extracted from natural images, movies, speech, or narratives and used in regression-based models to predict brain activity. Although naturalistic fMRI studies were initially centered on ISC, model-based approaches to naturalistic stimuli had already been developed in visual and auditory neurophysiology (Gallant et al., 1998; Theunissen et al., 2000; Wu et al., 2006). Early studies demonstrated that encoding models could identify natural images and movies from fMRI activity by modeling visual features in the stimulus (Kay et al., 2008; Naselaris et al., 2009; Nishimoto et al., 2011). This framework was later extended to higher-level semantic representations extracted from narratives and movies, revealing broad cortical maps of semantic selectivity (Huth et al., 2012, 2016; Çukur et al., 2013). Conversely, decoding models use brain activity as input to predict stimulus features, categories, or even reconstruct aspects of the perceived stimulus (Miyawaki et al., 2008; Nishimoto et al., 2011). Encoding and decoding models are complementary because the former predict brain activity from stimulus or model-derived features, whereas the latter infer stimulus-related information from brain activity (Naselaris et al., 2011). However, decodability indicates that measured activity patterns contain decoder-accessible information about the target variable; it does not necessarily show that this information is causally used in the relevant cognitive process (Kriegeskorte and Douglas, 2019).

At first glance, naturalistic paradigms may seem less hypothesis-driven than controlled paradigms because they do not typically rely on tightly designed contrasts to isolate target psychological constructs. Instead, participants are exposed to complex stimuli whose features are not fully manipulated in advance. This does not mean, however, that naturalistic paradigms are purely data-driven or hypothesis-free. In encoding-model approaches, theoretical assumptions enter through the choice of feature spaces used to describe the stimulus, behavior, or cognitive process of interest (Naselaris et al., 2011; Kriegeskorte and Douglas, 2019). For example, by choosing models based on low-level sensory features, linguistic features, or higher-level stimulus categories, researchers make different assumptions about which dimensions are relevant for explaining brain activity (Nishimoto et al., 2011; de Heer et al., 2017; Nakai et al., 2021a,b). This is also illustrated by studies using the same Cam-CAN movie data to examine different constructs, such as emotion (Majumdar et al., 2023), fluid intelligence (Petrican et al., 2021), and belief updates (Yazin et al., 2025), depending on the selected model or annotation space. These hypotheses can then be evaluated by comparing how well different feature spaces predict brain responses.

In ISC-based studies, theoretical assumptions can enter in a different way. Standard ISC identifies brain activity shared across participants exposed to the same stimulus (Hasson et al., 2004; Nastase et al., 2019), whereas extensions test whether neural similarity varies with participant-level characteristics or relationships, such as shared narrative interpretation, social network proximity, or political attitudes (Parkinson et al., 2018; Nguyen et al., 2019; Leong et al., 2020). In such cases, the explanatory variable is not a stimulus feature, but an inter-individual relation defined across participants. Theoretical assumptions therefore enter through participant selection, grouping, and the choice of relations used to explain neural similarity, rather than through the design of experimental contrasts alone.

Taken together, naturalistic paradigms do not eliminate theoretical assumptions; rather, they relocate them to the choice of stimulus feature spaces or participant-level relational variables. This shift allows researchers to study brain activity under richer and more ecologically valid conditions, while preserving the need for explicit assumptions about how psychological constructs are operationalized and interpreted.

### Computational modeling and NeuroAI approach

The use of naturalistic paradigms has been closely linked to the rise of computational modeling and the NeuroAI approach in cognitive neuroscience (Yamins and DiCarlo, 2016; Kriegeskorte and Douglas, 2018; Hasson et al., 2020). NeuroAI broadly refers to an interdisciplinary approach linking neuroscience and artificial intelligence, including both the use of artificial neural networks as models of brain data and the use of neuroscience-inspired principles to build more brain-like artificial agents (Zador et al., 2023). The present discussion mainly focuses on the former. In particular, encoding/decoding models can be combined with ANNs to relate brain activity to high-dimensional feature spaces learned from large-scale data (Güçlü and van Gerven, 2015; Horikawa and Kamitani, 2017; Schrimpf et al., 2021; Caucheteux et al., 2023; Nakai and Nishimoto, 2023a). Instead of relying on hand-crafted stimulus features, this approach uses internal representations from artificial neural networks as predictors of brain activity, allowing researchers to test whether model representations align with biological neural representations.

This shift has also changed the scale of neuroimaging data. Many computational modeling studies collect large amounts of data from each individual to train subject-specific predictive models, a strategy supported by recent studies showing that prediction improves with larger fMRI training sets (Antonello et al., 2023; Matsuyama et al., 2023). This contrasts with conventional group-level analyses, which often emphasize averaging across participants to identify effects that are consistent at the population level. This trend has been further accelerated by the growing availability of large-scale public brain datasets. For example, the Natural Scenes Dataset provides high-resolution 7 T fMRI responses from eight participants viewing tens of thousands of natural images, while the Narratives dataset aggregates hundreds of story-listening fMRI scans across diverse spoken narratives (Nastase et al., 2021; Allen et al., 2022). Together, these resources have enabled more systematic benchmarking of encoding models and have encouraged a shift from group-level localization of predefined effects toward within-subject predictive modeling of high-dimensional brain activity. However, extending such data-intensive within-subject modeling approaches to developmental and clinical populations, for whom long experimental sessions are often impractical, remains an important challenge.

This NeuroAI approach further changes where researchers place their assumptions. In controlled paradigms, theoretical assumptions are primarily embedded in the design of tasks and stimuli before data collection. Variables are therefore selected, simplified, or controlled at the level of experimental design. In naturalistic NeuroAI approaches, by contrast, many aspects of the stimulus and behavioral context are preserved during data acquisition, and variables are selected or constrained later through model design. The architecture of the ANN, the data on which it was trained, the objective function used for training, and the layer or representation selected for analysis all shape the feature space that is ultimately compared with brain activity (Yamins and DiCarlo, 2016; Kriegeskorte and Douglas, 2019; Lindsay, 2021; Yang and Wang, 2021). Thus, replacing hand-designed task contrasts with learned model representations does not remove theoretical assumptions. It shifts the locus of hypothesis testing from task and stimulus design to model specification, feature-space selection, and predictive evaluation.

### Overcome controlled vs. naturalistic dichotomy

Although naturalistic paradigms combined with AI-based models have greatly expanded cognitive neuroimaging, many studies still rely on passive viewing or listening with minimal task demands. Such designs are well suited for modeling perception, comprehension, and stimulus-driven semantic processing, but they capture only part of human cognition. A comprehensive account of brain function must also include active cognitive operations, such as problem solving, decision making, planning, and the manipulation of internal representations.

A promising direction is to integrate structured task design with computational modeling. Rather than choosing between highly controlled contrasts and unconstrained naturalistic stimulation, researchers can design tasks that systematically manipulate theoretically meaningful cognitive dimensions while retaining enough complexity to support model-based analysis. In such hybrid paradigms, experimental design specifies the broad structure of the cognitive space to be sampled, whereas encoding models, decoding models, or ANNs are used to predict brain activity within that space. This approach preserves the interpretability of controlled paradigms while benefiting from the flexibility and predictive power of naturalistic paradigms. It can also address the problem of generalization beyond narrowly tested conditions, which has been a major limitation of conventional controlled paradigms (Yarkoni, 2020).

This strategy has been illustrated in studies using large sets of active cognitive tasks. For example, (Nakai and Nishimoto, 2020) measured fMRI responses while participants performed 103 diverse cognitive tasks and constructed voxel-wise encoding and decoding models. These models revealed a hierarchical cortical organization of diverse cognitive functions and enabled prediction and decoding of novel task conditions. Subsequent work extended these analyses to the cerebellum and subcortex, showing that these regions exhibit task-related representational structures and decoding performance comparable to those observed in the cerebral cortex (Nakai and Nishimoto, 2022). Furthermore, representational geometry derived from encoding-model weights has been used to quantify individual differences in cognitive representations, revealing both subject-invariant and subject-specific structures (Nakai et al., 2025). These studies suggest that combining diverse active tasks with encoding models can contribute to a more comprehensive characterization of cognitive functions across the brain.

This approach can also be applied to specific cognitive domains that require active task engagement. Mathematical cognition is one such domain, involving symbolic manipulation, sequential reasoning, and problem solving that are difficult to elicit through passive naturalistic stimulation alone. Active task design can sample specific structures of mathematical operations, while computational models can characterize their neural representational geometry. For example, voxel-wise encoding models have revealed format-invariant representations of mathematical problems (Nakai and Nishimoto, 2023b), and alignment between neural and ANN representations of symbolic mathematical operations (Nakai and Nishimoto, 2023a).

Games and virtual reality provide another promising approach that bridge controlled and naturalistic paradigms. They allow researchers to study rich, active cognitive processes, such as exploration, learning, planning, and policy selection, while preserving experimental control over task structure and environmental dynamics (Tomov et al., 2023; Allen et al., 2024; Haberland et al., 2026). For example, Atari-style video games have been combined with theory-based reinforcement-learning models to examine how brain responses reflect latent processes such as theory formation and prediction errors during interactive learning (Tomov et al., 2023).

Together, these findings illustrate how active, hypothesis-guided tasks can be combined with computational models to study cognitive domains that are difficult to capture through passive naturalistic stimulation alone. More broadly, they suggest that the future of cognitive neuroimaging lies not in replacing controlled paradigms with naturalistic ones, but in integrating the theoretical precision of controlled task design with the richness and predictive power of computational modeling.

## Conclusion

In conclusion, controlled and naturalistic paradigms should not be viewed as competing alternatives, but as complementary strategies for linking psychological constructs to brain activity. Controlled paradigms provide interpretability through carefully designed tasks and experimental contrasts, whereas naturalistic paradigms, especially when combined with computational modeling and NeuroAI approaches, preserve richer stimulus structure and enable brain activity to be modeled in relation to continuous features of real-world inputs. Future cognitive neuroscience should therefore integrate the precision of controlled experimentation with the richness and predictive power of naturalistic modeling. Such integration would make it possible to extend computational modeling beyond passive viewing and listening to active cognition.

## Data Availability

The original contributions presented in the study are included in the article/supplementary material, further inquiries can be directed to the corresponding author.
